# Ohio State Dental Society

**Published:** 1870-12

**Authors:** 


### OHIO STATE DENTAL SOCIETY.

The late meeting of our State Society was, in many respects, the most interesting ever held by it. The attendance was better than ever before; attention to business was more manifest, the discussions were more earnest and scientific than usual, the papers read received closer attention than is commonly awarded to such documents, the membership of the Society was greatly increased, the utmost harmony and good feeling prevailed, and really it was a good meeting to attend. Those who failed to be there will never know how much they have lost; and their patrons will, for a time, remain in like blissful ignorance; but, as light diffuses, patients will learn that it is neither safe nor satisfactory to trust the care of their mouths to those who neglect such opportunities for professional advancement.

Some sixty or seventy members were present, including many of the most earnest and experienced workers in the profession. Then, too, so many live young men were present—active, earnest, thinking, working—that even the prince of croakers can not despond in reference to the future of Dentistry in Ohio.

The Ohio State Dental Society is one of the most important Dental Associations. It elects and supports the State Board of Dental Examiners, and thus is responsible for the faithful execution of the law regulating the practice of Dentistry in the State. In view of this responsibility, at the late meeting, it was resolved by a unanimous vote, that non-graduates are not eligible for membership without a certificate from the Board. Such a measure was necessary to the safety of the profession.

The beneficial results of the law are already clearly manifest. The five years allowed to those already in practice are being eagerly improved by the younger and more active members who possess the ambition necessary to the proper practice of any profession. Many of these began careful, systematic study as soon sa the law passed; and a number of them have come forward and received certificates from the Board. How much their patients have been blessed by their increased knowledge of dental science, no one can estimate. Quite a number of notorious quacks have been prevented from settling in the State in view of the law requiring a certificate. These have either abandoned the practice, or sought locations not subject to such restriction. Some, too, already in practice, conscious of their unfitness and unable or unwilling to qualify themselves, are making arrangements to follow more congenial pursuits, where they will be far more useful to society and themselves, from the fact of their being better qualified to discharge their duties. In this way, while society is benefited, the profession is elevated.

Much of the success of the late meeting is due to the common sense and ability with which Dr. Rehwinkle presided; and in view of the fact that Dr. Geo. W. Keely is his successor, there will not likely be any discounting of the next meeting. Dr. Herriott was re-elected Recording Secretary, and Geo. Watt was re-elected Treasurer. (Of course, members in arrears will know where to send their annual dues.) Dr. C. R. Butler is Corresponding Secretary; and for the Vice Presidents and standing committees we must refer the reader to the proceedings to be published hereafter.

And now, reader, you had best be on hand yourself next December; for you can't keep up with the progress of the profession without attending the meetings of the Ohio State Dental Society. W.

